# Supplementary material for: Adapting Machine Learning Diagnostic Models to New Populations Using a Small Amount of Data: Results from Clinical Neuroscience
Source: ArXiv. 2024 Sep 13:arXiv:2308.03175v2. Preprint. [Version 2] (PMC11419182)
Supplement: Supplement 1 [file NIHPP2308.03175v2-supplement-1.pdf]

A Additional Tables and Figures

Table S.1: Summary of the data from the iSTAGING consortium used for early diagnosis of stable and progressive mild cognitive impairment.

**Mild Cognitive Impairment**		ADNI-1 (44.60%)	ADNI-2/3 (47.69%)	PENN (2.31%)	AIBL (5.40%)	Total
Subjects						
	Progressive MCI	197	113	5	12	327
	Stable MCI	79	151	10	11	251
	Normal MCI	13	45	-	12	70
Sex (%)						
	Female	15.90	21.30	1.08	2.01	40.28
	Male	28.70	26.39	1.23	3.40	59.72
Age (%, years)						
	0–65	5.86	10.80	0.62	0.15	17.44
	65–70	5.86	11.27	0.46	1.39	18.98
	70–75	11.57	11.73	0.46	1.85	25.62
	75–80	10.19	11.73	0.62	1.39	21.60
	> 80	11.11	4.48	0.15	0.62	16.36
Race (%)						
	White	42.75	43.83	2.16	2.16	90.90
	Black	0.77	0.93	0.15	-	1.85
	Asian	1.08	0.62	-	-	1.70

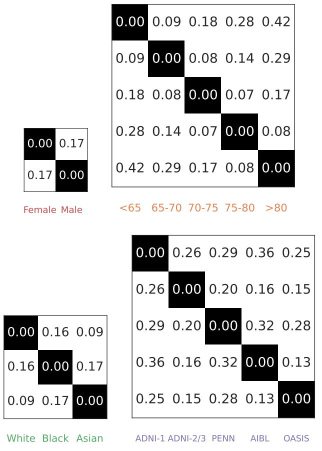

(a)

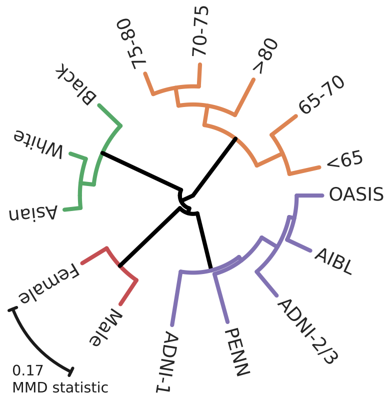

(b)

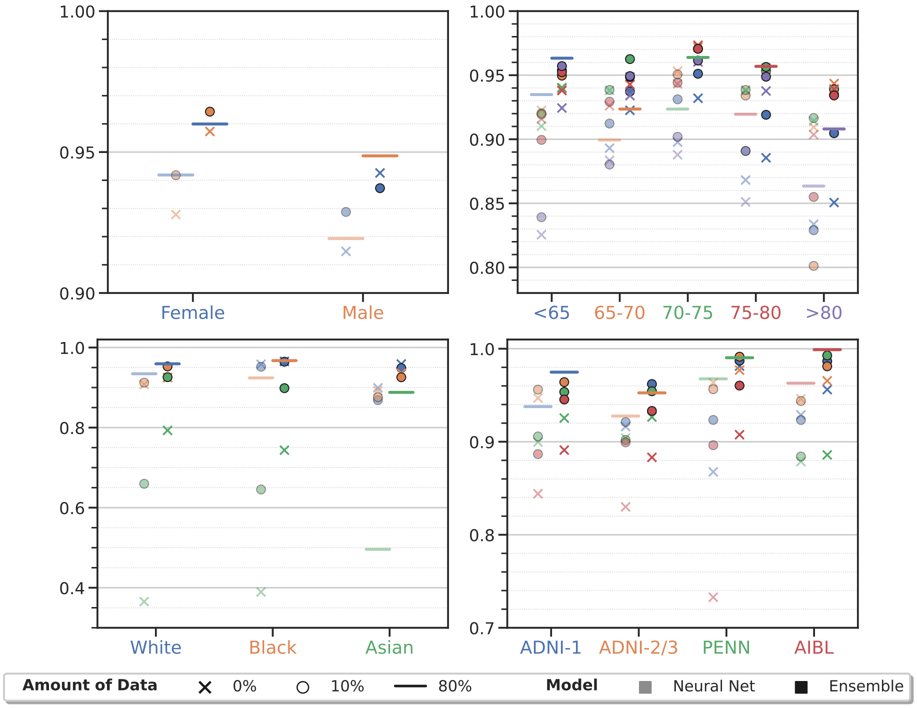

(c)

Figure S.1: Discrepancy in the data and AUC of diagnostic models of Alzheimer's disease on different groups in the population. (a-b) Distance between leaves of this dendrogram in **(b)** indicates the pairwise MMD statistic in **(a)** between learned features of pairs of groups, e.g., distributional discrepancy between Male-Female groups is 0.17, while the distributional discrepancy between < 65 years and > 80 years, or between ADNI-1 and ADNI-2/3, is larger (0.42 and 0.26 respectively). **(c)** Average AUC of Alzheimer's disease classification computed using five-fold nested cross-validation. We trained a machine learning model, either a deep neural network (translucent markers) or an ensemble using boosting, bagging and stacking (bold markers), using data from different source groups (different colors) and evaluated this model (cross marks) on data from different target groups (X-axis); circles denote model fitted using our α-weighted empirical risk minimization (ERM) procedure with access to 10% data from the target group; horizontal lines denote models that are directly trained on the target group using 80% of data (the rest for testing). All models use data from multiple sources, namely structural measures, demographic, clinical variables, genetic factors, and cognitive scores.

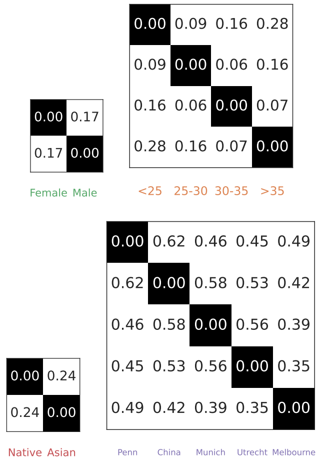

(a)

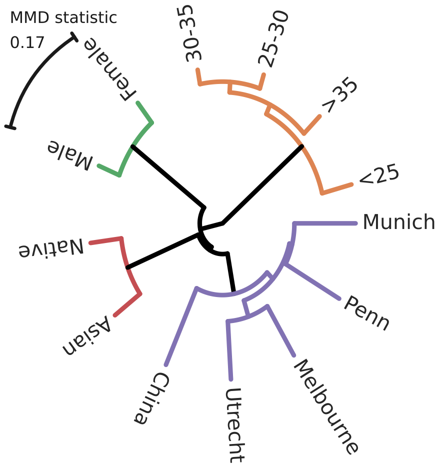

(b)

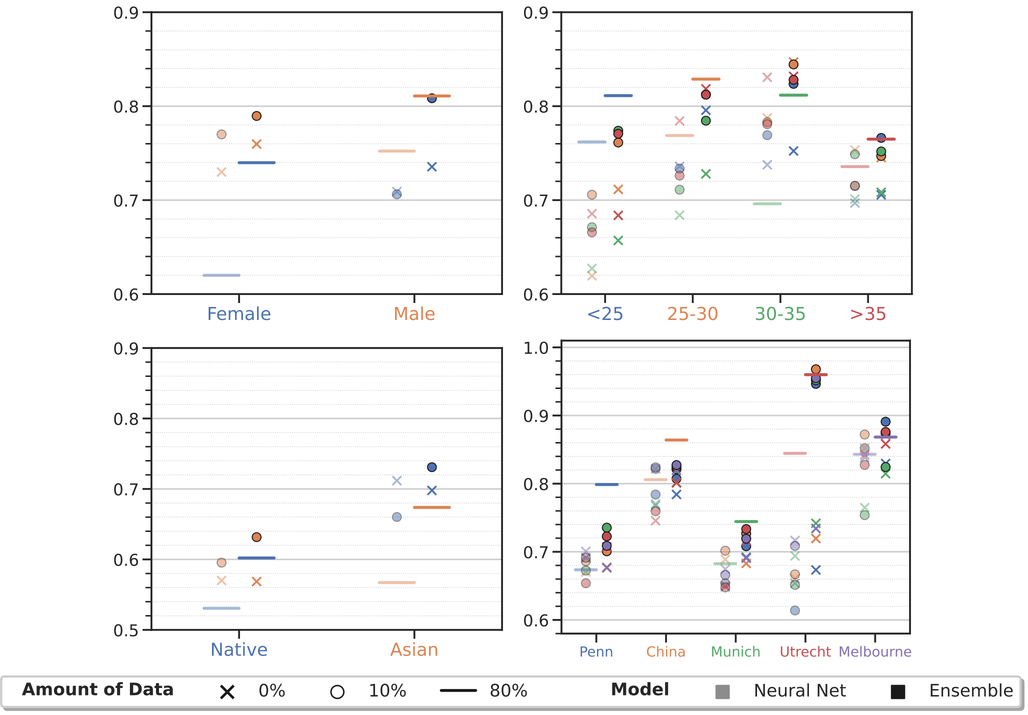

(c)

Figure S.2: Discrepancy in the data and AUC of diagnostic models of schizophrenia on different groups in the population. (a-b) Distance between leaves of this dendrogram in **(b)** indicates the pairwise MMD statistic in **(a)** between learned features of pairs of groups. **(c)** Average AUC of schizophrenia classification computed using five-fold nested cross-validation. We trained a deep neural network (translucent markers) and an ensemble using boosting, bagging and stacking (bold markers), using data from different source groups (different colors) and evaluated this model (cross marks) on data from different target groups (X-axis); circles denote model fitted using our α-weighted empirical risk minimization (ERM) procedure with access to 10% data from the target group; horizontal lines denote models that are directly trained on the target group using 80% of data (the rest for testing). Similar to diagnostic models of Alzheimer's disease, in general, (i) the AUC of ensemble models is higher than that of the neural network in all cases ($p < 0.01$), (ii) AUC of a model trained on a source group remains remarkably high when evaluated on the target group (crosses), (iii) in almost all cases for the ensemble, it further improves when one has access to a small fraction of data from the target group (circles are higher than crosses), and (iv) some times even beyond the AUC of a model trained only on the target group (circles above horizontal lines).

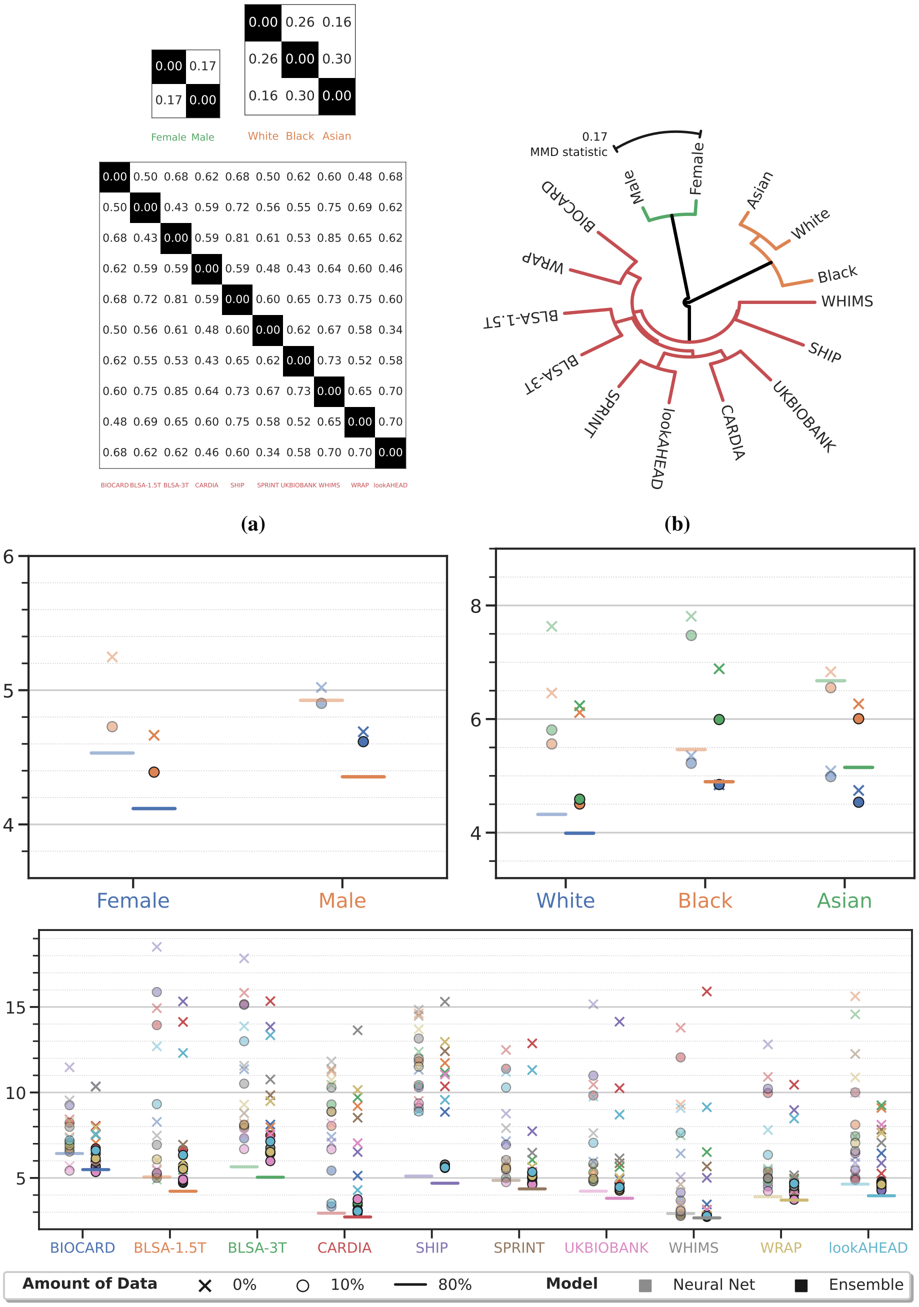

(c)

Figure S.3: Discrepancy in the data and MAE (in years) models that predict the brain age. (a-b) Distance between leaves of this dendrogram in **(b)** indicates the pairwise MMD statistic in **(a)** between learned features of pairs of groups. **(c)** Average AUC of schizophrenia classification computed using five-fold nested cross-validation. We trained a deep neural network (translucent markers) and an ensemble using boosting, bagging and stacking (bold markers), using data from different source groups (different colors) and evaluated this model (cross marks) on data from different target groups (X-axis); circles denote model fitted using our α-weighted empirical risk minimization (ERM) procedure with access to 10% data from the target group; horizontal lines denote models that are directly trained on the target group using 80% of data (the rest for testing). Similar to diagnostic models of Alzheimer's disease and schizophrenia, in general, (i) the MAE of ensemble models is lower than that of the neural network in all cases ($p < 0.01$), and (ii) in almost all cases for the ensemble, the MAE improves when one has access to a small fraction of data from the target group (circles are lower than crosses).

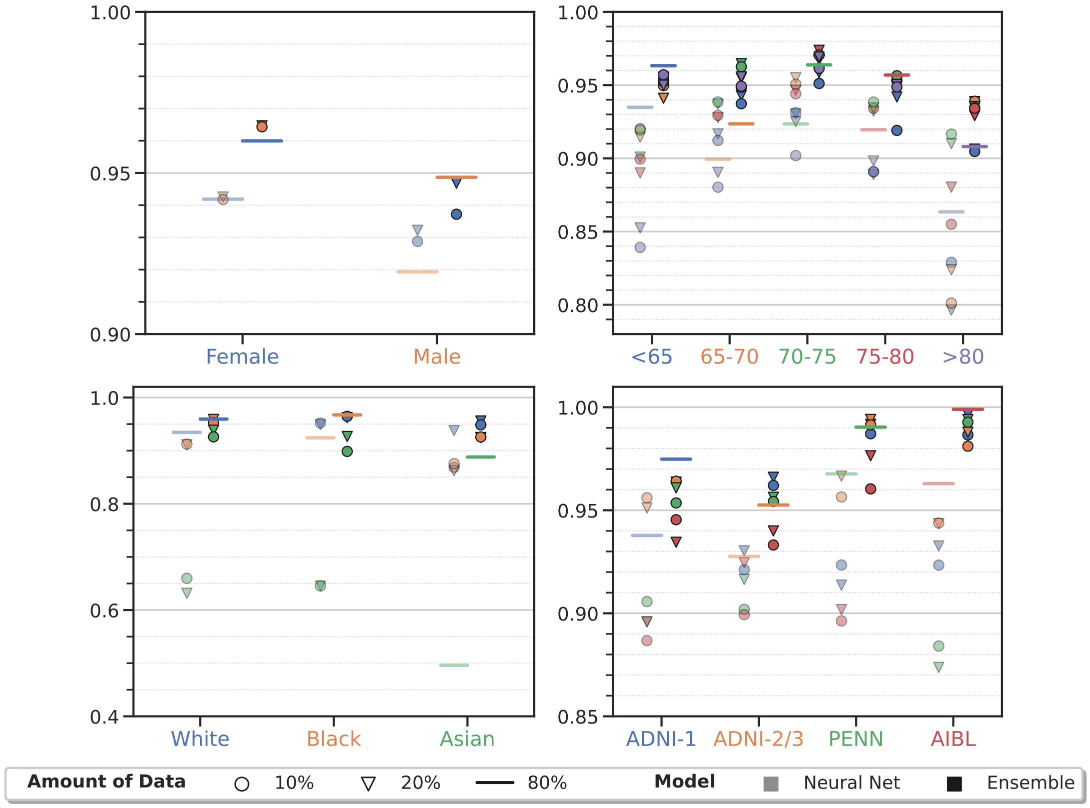

Figure S.4: Average AUC of Alzheimer's disease classification computed using five-fold nested cross-validation. We trained a machine learning model, either a deep neural network (translucent markers) or an ensemble using boosting, bagging and stacking (bold markers), using data from different source groups (different colors); circles and triangles denote model fitted using our α-weighted empirical risk minimization (ERM) procedure with access to 10% and 20% data respectively from the target group; horizontal lines denote models that are directly trained on the target group using 80% of data (the rest for testing). All models use data from multiple sources, namely structural measures, demographic, clinical variables, genetic factors, and cognitive scores. In some cases, the AUC using 20% data is better (triangles above circles) but overall the AUC of models trained with 20% target data is statistically the same as that of using 10% data. This indicates that we can fruitfully predict the Alzheimer's disease diagnosis for subjects from different groups in the population with access to as little as 10% data from those groups.

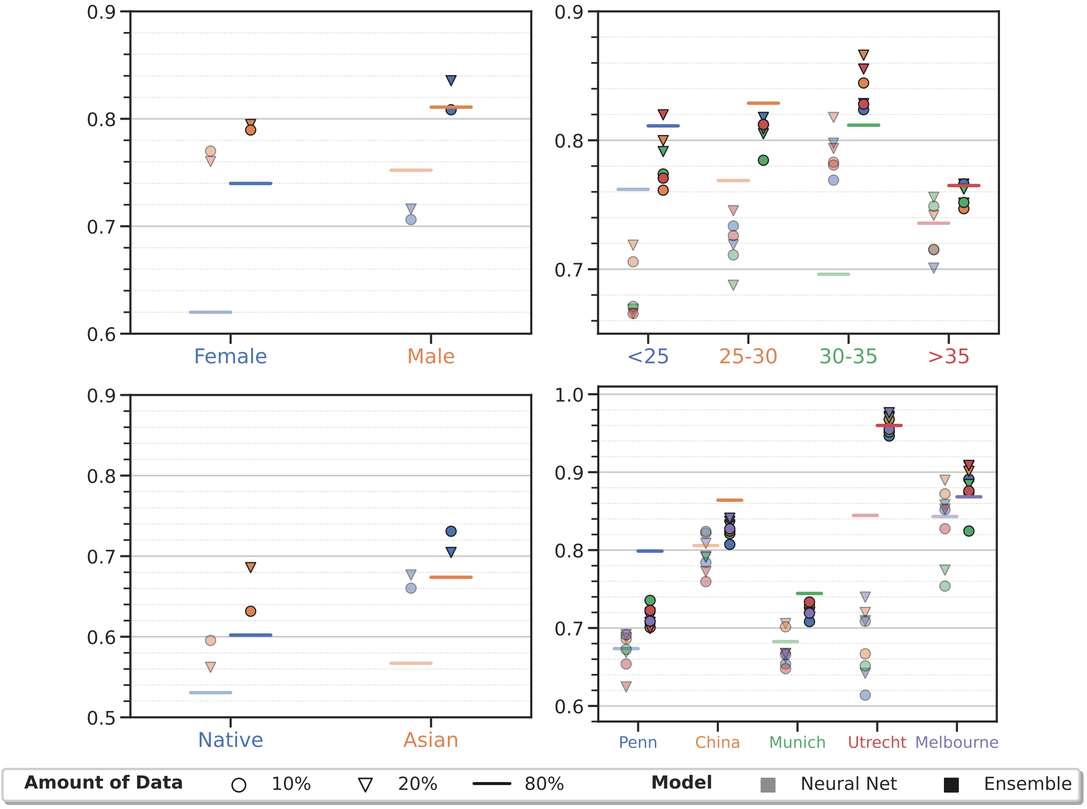

Figure S.5: Average AUC of schizophrenia classification computed using five-fold nested cross-validation. We trained a machine learning model, either a deep neural network (translucent markers) or an ensemble using boosting, bagging and stacking (bold markers), using data from different source groups (different colors); circles and triangles denote model fitted using our α-weighted empirical risk minimization (ERM) procedure with access to 10% and 20% data respectively from the target group; horizontal lines denote models that are directly trained on the target group using 80% of data (the rest for testing). All models use data from multiple sources, namely structural measures, demographic, clinical variables, genetic factors, and cognitive scores. In some cases, the AUC using 20% data is better (triangles above circles) but overall the AUC of models trained with 20% target data is statistically the same as those trained with 10% data. This indicates that we can fruitfully predict the schizophrenia diagnosis for subjects from different groups in the population with access to as little as 10% data from those groups.

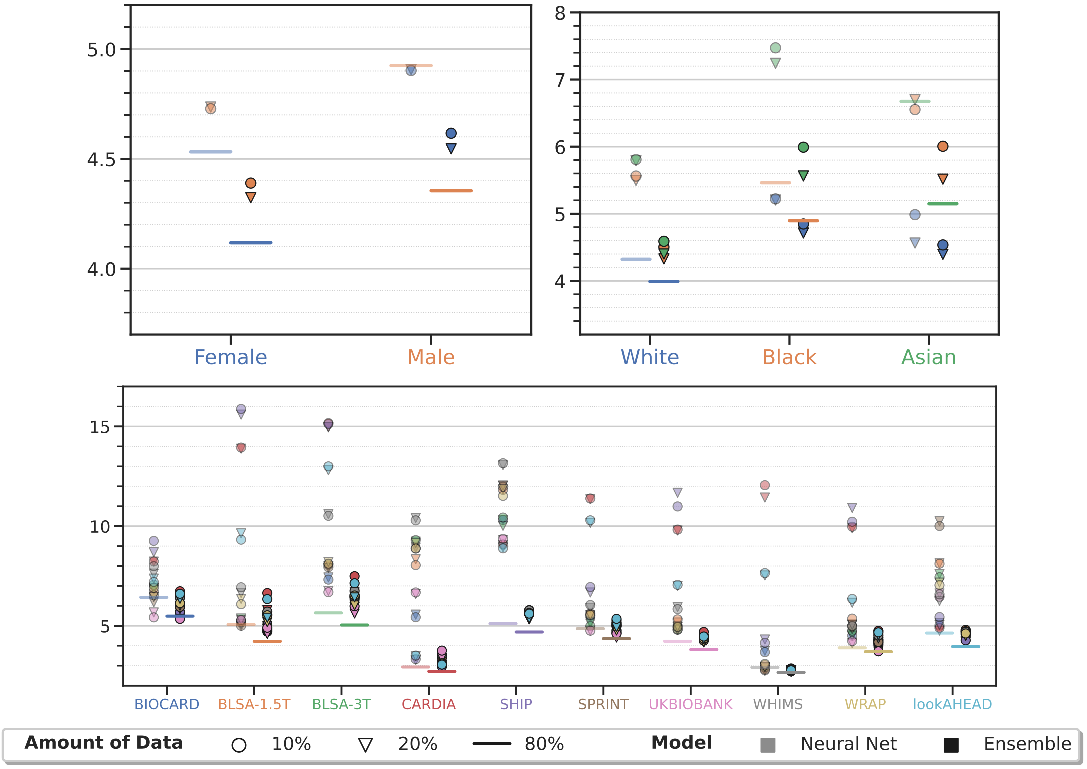

Figure S.6: Average mean average error (MAE) in years of predicting the brain age, computed using five-fold nested cross-validation. The setup is the same as that of Figs. S.4 and S.5. For brain age prediction, again, we see that the MAE obtained using 20% data is some times better than of 10% data (triangles below circles) but overall the two settings achieve the same MAE statistically. This indicates that we can fruitfully predict the brain age of subjects from different groups in the population with access to as little as 10% data from those groups.

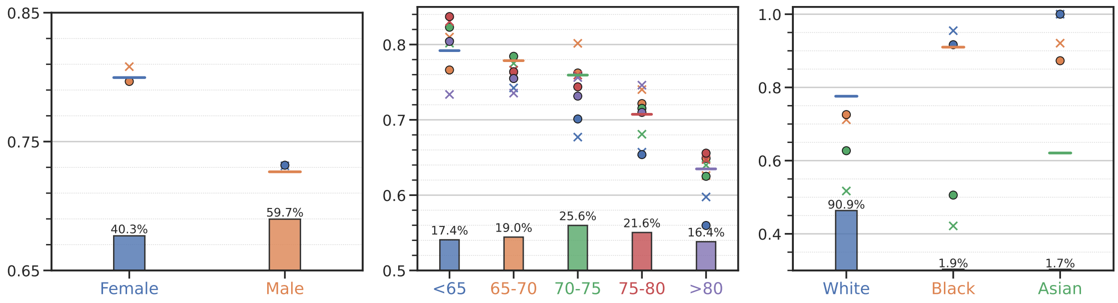

(a)

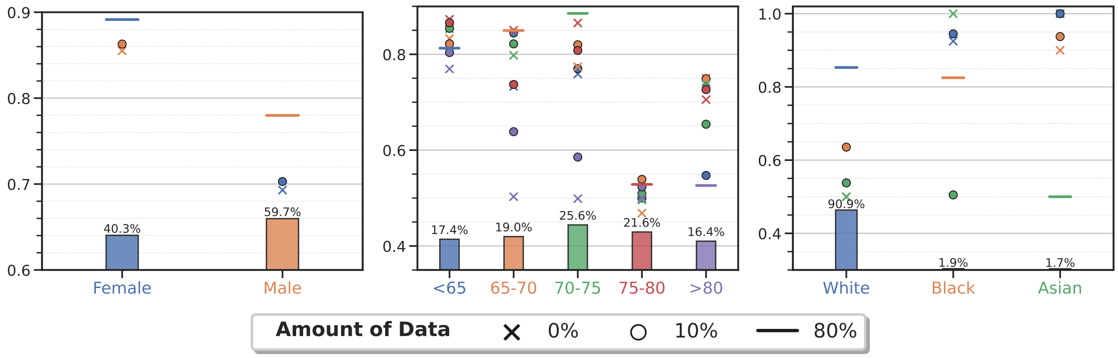

(b)

Figure S.7: Linear discriminant analysis on the output probabilities (that determines AD vs. control) of the ensemble models trained for Alzheimer's disease diagnosis is used to study whether subjects with mild cognitive impairment (MCI) progress to AD (known as pMCI) or remain stable MCI (known as sMCI) in **(a)**, and pMCI vs. nMCI in **(b)** where the latter refers to MCI subjects diagnosed as normal within three years of the first clinical visit. The AUC of pMCI vs. sMCI on the target group is shown for three different attributes (sex, age group and race) when models are trained only on data from the source group (crosses), using α-weighted ERM using all data from the source and 10% data from the target group (circles) and with access to only all data from the target group (horizontal lines). Improvements in the AD vs. control AUC of these models with 10% data translate to improvements in the ability to distinguish between pMCI and sMCI subjects, using only baseline scans (circles above cross) except when target groups are Black or Asian (due to very little data in these groups).

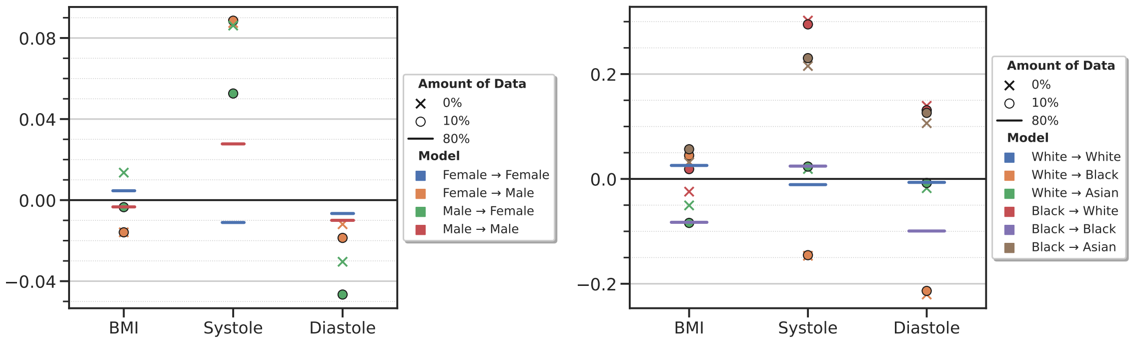

Figure S.8: Correlation of brain age regression with cardiovascular and lifestyle factors. Pearson's correlation between the brain age residual (predicted brain age minus chronological age) and clinical factors for two different attributes (sex and race) for models trained only on source data (crosses), using α-weighted ERM on all source data and 10% target data (circles) and only on all target data (horizontal lines). Unlike other plots, colors denote different pairs of source and target groups. Tests (X-axis) marked in red are expected to be negatively correlated with brain aging whereas those marked in black are expected to be positively correlated with brain aging according to the existing literature. Body mass index (BMI) is an individual's health indicator based on tissue mass (muscle, fat, and bone) and height. Systolic and diastolic blood pressures are the measures of heart functioning. In almost all cases, we observe weak correlations which are statistically insignificant (p-value > 0.01).

Table S.2: Summary of variables in the data from the iSTAGING consortium (Alzheimer's disease and brain age) and the PHENOM consortium (schizophrenia) used in this study. The numbers next to the variable names are the dimensionality.

**Variables**			iSTAGING	PHENOM
MR imaging				
	Region-of-interest volumes	145	✓	✓
	White matter lesion volume	1	✓	
Demographics				
	Gender	1	✓	✓
	Age	1	✓	✓
	Race	1	✓	✓
	Education level	1		✓
	Marital status	1		✓
	Employment status	1		✓
	Handedness	1		✓
	Smoking status	1	✓	
Clinical				
	Diabetes	1	✓	
	Hypertension	1	✓	
	Hyperlipidemia	1	✓	
	Blood pressure (systolic/diastolic)	2	✓	
	Body mass index	1	✓	
Genetic factor				
	Apolipoprotein E allele 2	1	✓	
	Apolipoprotein E allele 3	1	✓	
	Apolipoprotein E allele 4	1	✓	
Cognitive score				
	Mini-mental state exam	1	✓	

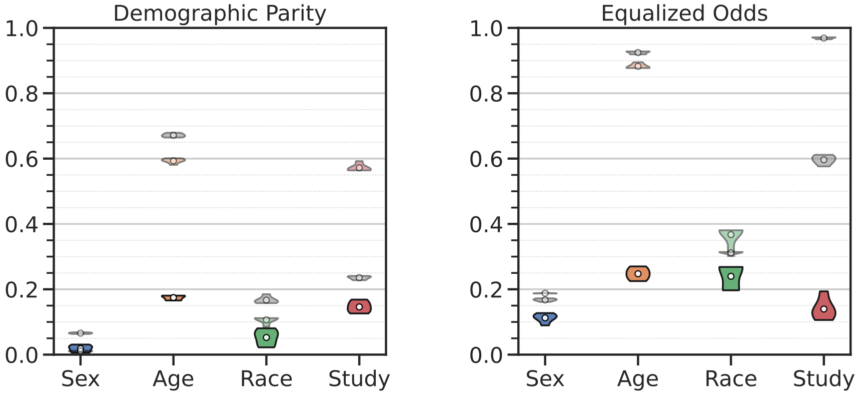

(a) Alzheimer's disease

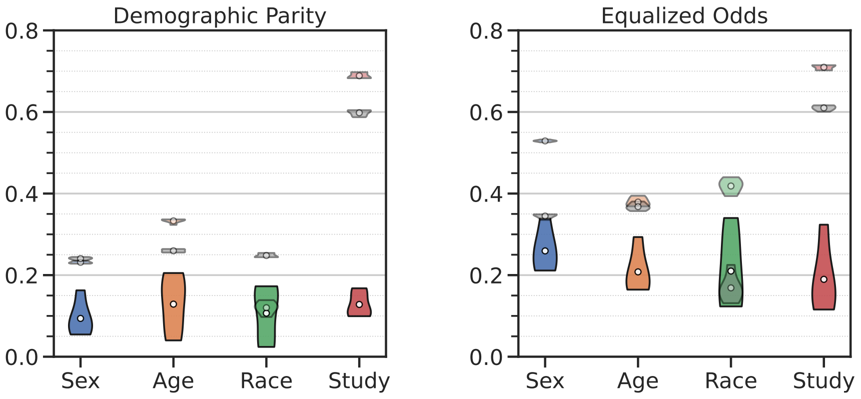

(b) Schizophrenia

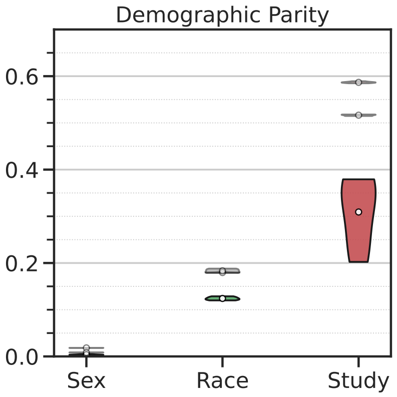

(c) Brain age prediction

Figure S.9: Fairness assessment of machine learning models with respect to sensitive attributes including sex, age group, race, and clinical study. Violin plots denote the test demographic parity differences (DPD) and equalized odds difference (EOD) on five different held-out subsets of data. Translucent gray denotes the performance of a baseline deep network while translucent colors indicate the ensemble models. Solid colors indicate using α-weighted ERM on all source data and 10% target data. White dots denote the average performance of each group. A model is perfectly fair if the fairness disparity (DPD or EOD) is zero. In general, we observe that our α-weighted ERM models have lower disparities for all sensitive attributes in both metrics compared to the neural net and ensemble models.

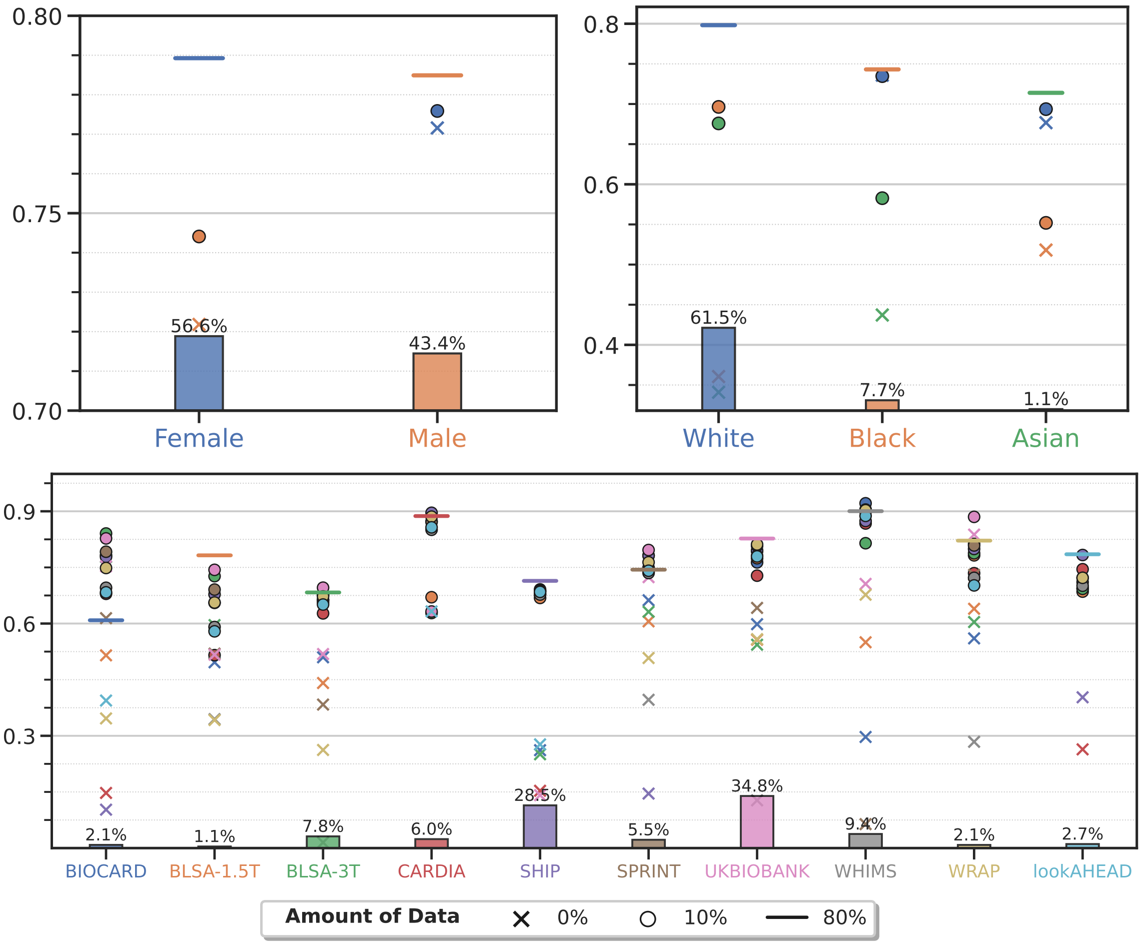

Figure S.10: Brain age prediction with coefficient of determination metric. Markers denote the coefficient of determination (R^2) of an ensemble that predicts the brain age on different target groups in the population computed using five-fold nested cross-validation, for models trained only on data from the target group (e.g., Female subjects, denoted by the blue horizontal line), only on data from the source group (crosses), and trained on all data from the source group and 10% data from the target group (circles). we find that using R^2 agrees with our original conclusions drawn from MAE for brain age prediction. Note that R^2 can be negative on the test data; this is important when a model trained on one group, say age group, is being evaluated on another where subjects are from a different age group. We have therefore truncated our plot at zero. A baseline model (crosses) cannot generalize well to a new out-of-distribution group in general, whereas our weighted-ERM (circles in the figures) improves the performance significantly. It should also be noted that many baseline models (crosses) have negative R^2 scores in the cross-study scenarios (lower panel). Our proposed approach that uses a small amount of target data for adaptation (circles in the figures) has consistently high R^2 scores.

Table S.3: Summary of the data from the iSTAGING consortium (Alzheimer's disease and brain age) and the PHENOM consortium (schizophrenia) used in this study.

**Alzheimer's Disease**		ADNI-1 (17.37%)	ADNI-2/3 (23.29%)	PENN (25.44%)	AIBL (10.07%)	OASIS (23.82%)	Total
Subjects							
	Control	173	261	228	119	276	1057
	Patient	191	227	305	92	223	1038
Sex							
	Female	174	250	340	129	266	1160
	Male	190	238	192	82	233	935
Age (years)							
	0–65	24	56	101	29	103	312
	65–70	31	130	112	41	107	421
	70–75	116	111	109	60	95	491
	75–80	104	110	105	41	101	461
	> 80	89	81	106	40	93	409
Race							
	White	337	271	401	117	409	1537
	Black	19	14	107	-	85	225
	Asian	5	9	10	-	4	28

**Schizophrenia**		Penn (22.28%)	China (13.94%)	Munich (29.64%)	Utrecht (20.12%)	Melbourne (14.03%)	Total
Subjects							
	Control	131	76	157	115	84	563
	Patient	96	66	145	90	59	456
Sex							
	Female	121	69	79	71	41	381
	Male	106	73	223	134	102	638
Age (years)							
	0–25	59	50	96	103	60	368
	25–30	64	24	74	42	22	226
	30–35	36	22	61	29	14	162
	> 35	68	46	71	31	47	263
Race							
	Native	107	-	-	-	-	107
	Asian	75	-	-	-	-	75

**Brain Age**		BIOCARD (2.12%)	BLSA-1.5T (1.05%)	BLSA-3T (7.82%)	CARDIA (5.97%)	SHIP (28.54%)	SPRINT (5.54%)	UKBB (34.79%)	WHIMS (9.40%)	WRAP (2.09%)	lookAHEAD (2.68%)	Total
Subjects												
	Control	246	122	907	693	3311	643	4036	1090	242	311	11601
Sex												
	Female	152	53	506	344	1695	227	2101	1081	169	222	6562
	Male	94	68	400	348	1609	416	1931	-	73	90	5039
Age (years)												
	Mean	57.61	67.87	64.06	51.00	52.81	68.58	62.81	69.59	63.57	58.05	60.14
	Min	21	48	22	42	21	50	45	64	50	44	21
	Max	86	85	92	61	90	91	79	79	78	74	92
Race												
	White	243	111	597	388	-	420	3915	1003	230	227	7133
	Black	2	11	229	304	-	202	24	46	5	70	899
	Asian	-	-	57	-	-	6	48	15	1	-	126

Table S.4: Summary of the data from the iSTAGING consortium used for early diagnosis of stable and progressive mild cognitive impairment.

**Mild Cognitive Impairment**		ADNI-1 (44.60%)	ADNI-2/3 (47.69%)	PENN (2.31%)	AIBL (5.40%)	Total
Subjects						
	Progressive MCI	197	113	5	12	327
	Stable MCI	79	151	10	11	251
	Normal MCI	13	45	-	12	70
Sex						
	Female	103	138	7	13	261
	Male	186	171	8	22	387
Age (years)						
	0–65	38	70	4	1	113
	65–70	38	73	3	9	123
	70–75	75	76	3	12	166
	75–80	66	76	4	9	140
	> 80	72	29	1	4	106
Race						
	White	277	283	14	14	589
	Black	5	6	1	-	12
	Asian	7	4	-	-	11

Table S.5: Summary of the data from the iSTAGING consortium used for association study between the brain age residual and neuropsychological tests. Mini-mental state examination (MMSE) is a questionnaire test that measures global cognitive impairment. Digit span forward/backward (DSF/B) test is a way of measuring the storage capacity of a person's working memory. Trail making test part A/B (TMT A/B) measures a person's executive functioning. Digit symbol substitution test (DSST) is another global measure of cognitive ability, requiring multiple cognitive domains to complete effectively.

**Neuropsychological Test**		MMSE	DSF	TMT A	DSST	TMT B	DSB
Subjects							
	Control	491	1822	1741	1547	1724	595
Sex (%)							
	Female	60.08	56.70	57.04	55.07	56.90	56.13
	Male	39.92	43.30	42.96	44.93	43.10	43.87
Age (years)							
	Mean	62.05	61.46	61.48	58.98	61.43	60.95
	Min	22	22	22	43	22	22
	Max	91	91	91	87	91	91
Race (%)							
	White	70.47	88.42	88.00	88.30	87.88	69.92
	Black	18.94	6.92	7.29	10.28	7.37	19.83
	Asian	6.72	2.47	2.53	0.52	2.55	6.05

Table S.6: Summary of missing value sample size for each variable in the data from the iSTAGING consortium (Alzheimer's disease and brain age) and the PHENOM consortium (schizophrenia) used in this study.

**Variables**		Alzheimer's	Schizophrenia	Brain Age
MR imaging				
	Region-of-interest volumes	0	0	0
	White matter lesion volume	706	-	374
Demographics				
	Gender	0	0	0
	Age	0	0	0
	Race	287	820	3323
	Education level	-	566	-
	Marital status	-	789	-
	Employment status	-	783	-
	Handedness	-	565	-
	Smoking status	-	-	3694
Clinical				
	Diabetes	-	-	2389
	Hypertension	-	-	3826
	Hyperlipidemia	-	-	5616
	Blood pressure (systolic/diastolic)	-	-	3457
	Body mass index	-	-	3465
Genetic factor				
	Apolipoprotein E allele 2	550	-	5301
	Apolipoprotein E allele 3	550	-	5301
	Apolipoprotein E allele 4	550	-	5301
Cognitive score				
	Mini-mental state exam	1027	-	-

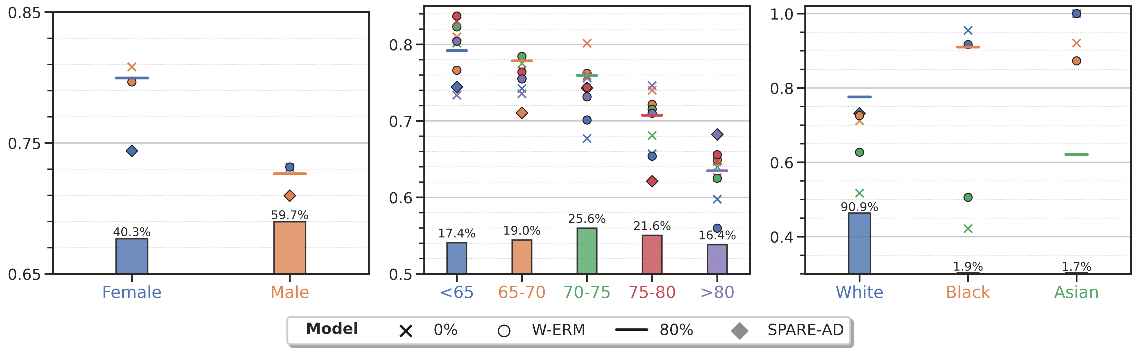

Figure S.11: Early diagnosis of stable and progressive mild cognitive impairment. Linear discriminant analysis on the output probabilities (that determines AD vs. cognitively normal CN) of the ensemble models trained for Alzheimer's disease diagnosis is used to study whether subjects with mild cognitive impairment (MCI) progress to AD (known as pMCI) or remain stable MCI (known as sMCI) using only the baseline scans. The AUC of pMCI vs. sMCI on the target group is shown for three different attributes (sex, age group and race) when models are trained only on data from the source group (crosses), using α-weighted ERM using all data from the source and 10% data from the target group (circles) and with access to only all data from the target group (horizontal lines). We also train models using SPARE-AD index instead of AD-probabilities from the target group for comparison (diamonds).

Table S.7: Alzheimer's disease classification results. We report the average AUC on the target group (columns in the tables) computed using five-fold nested cross-validation for models trained only on data from the target group (denoted by 80% in the tables), only on data from the source group (denoted by 0% in the tables), and trained on all data from the source group and 10% data from the target group (denoted by 10% in the tables). Panels denote groups stratified by one of the four attributes, namely sex, age group, race and clinical study. Bar plots denote the proportion of subjects in these groups in our study. All models are ensembles trained using features derived from structural measures, demographic and clinical variables, genetic factors, and cognitive scores.

**Sex**		Female	Male
0%			
	Female	-	0.9426
	Male	0.9573	-
10%			
	Female	-	0.9372
	Male	0.9643	-
80%			
	-	0.96	0.9487

**Age**		0-65	65-70	70-75	75-80	>80
0%						
	0-65	-	0.9226	0.9320	0.8855	0.8506
	65-70	0.9396	-	0.9718	0.9526	0.9435
	70-75	0.9403	0.9485	-	0.9555	0.9381
	75-80	0.9380	0.9428	0.9734	-	0.9392
	>80	0.9244	0.9341	0.9604	0.9377	-
10%						
	0-65	-	0.9373	0.9511	0.9191	0.9047
	65-70	0.9496	-	0.9707	0.9531	0.9390
	70-75	0.9537	0.9626	-	0.9564	0.9346
	75-80	0.9523	0.9485	0.9706	-	0.9342
	>80	0.9571	0.9493	0.9614	0.9488	-
80%						
	-	0.9633	0.9236	0.9638	0.9569	0.9080

**Race**		White	Black	Asian
0%				
	White	-	0.9653	0.9588
	Black	0.9251	-	0.9358
	Asian	0.7930	0.7437	-
10%				
	White	-	0.9646	0.9486
	Black	0.9528	-	0.9257
	Asian	0.9259	0.8985	-
80%				
	-	0.9594	0.9673	0.8880

**Study**		ADNI-1	ADNI-2/3	PENN	AIBL
0%					
	ADNI-1	-	0.9567	0.9814	0.9561
	ADNI-2/3	0.9496	-	0.9770	0.9655
	PENN	0.9255	0.9266	-	0.8858
	AIBL	0.8910	0.8832	0.9075	-
10%					
	ADNI-1	-	0.9621	0.9871	0.9866
	ADNI-2/3	0.9641	-	0.9914	0.9811
	PENN	0.9536	0.9542	-	0.9928
	AIBL	0.9454	0.9331	0.9604	-
80%					
	-	0.9748	0.9526	0.9903	0.9990

Table S.8: Schizophrenia classification results. We report the average AUC on the target group (columns in the tables) computed using five-fold nested cross-validation for models trained only on data from the target group (denoted by 80% in the tables), only on data from the source group (denoted by 0% in the tables), and trained on all data from the source group and 10% data from the target group (denoted by 10% in the tables). Panels denote groups stratified by one of the four attributes, namely sex, age group, race and clinical study. Bar plots denote the proportion of subjects in these groups in our study. All models are ensembles trained using features derived from structural measures, demographic and clinical variables, genetic factors, and cognitive scores.

**Sex**		Female	Male
0%			
	Female	-	0.7355
	Male	0.7597	-
10%			
	Female	-	0.8084
	Male	0.7896	-
80%			
	-	0.7398	0.8109

**Age**		0-25	25-30	30-35	>35
0%					
	0-25	-	0.7956	0.7523	0.7056
	25-30	0.7114	-	0.8469	0.7453
	30-35	0.6571	0.7279	-	0.7084
	>35	0.6838	0.8184	0.8318	-
10%					
	0-25	-	0.8123	0.8237	0.7662
	25-30	0.7613	-	0.8445	0.7469
	30-35	0.7739	0.7845	-	0.7518
	>35	0.7706	0.8122	0.8281	-
80%					
	-	0.8112	0.8288	0.8117	0.7649

**Race**		Native	Asian
0%			
	Native	-	0.6979
	Asian	0.5686	-
10%			
	Native	-	0.7309
	Asian	0.6316	-
80%			
	-	0.6020	0.6738

**Study**		Penn	China	Munich	Utrecht	Melbourne
0%						
	Penn	-	0.7842	0.6920	0.6734	0.8296
	China	0.6763	-	0.6829	0.7195	0.8714
	Munich	0.7279	0.8010	-	0.7418	0.8146
	Utrecht	0.7070	0.8016	0.7164	-	0.8583
	Melbourne	0.6771	0.8196	0.6907	0.7342	-
10%						
	Penn	-	0.8073	0.7082	0.9464	0.8909
	China	0.7007	-	0.7267	0.9679	0.8737
	Munich	0.7354	0.8211	-	0.9516	0.8244
	Utrecht	0.7226	0.8243	0.7334	-	0.8759
	Melbourne	0.7088	0.8273	0.7191	0.9548	-
80%						
	-	0.7987	0.8640	0.7444	0.9599	0.8684

Table S.9: Brain age prediction results. We report the average mean absolute error (MAE) in years on the target group (columns in the tables) computed using five-fold nested cross-validation for models trained only on data from the target group (denoted by 80% in the tables), only on data from the source group (denoted by 0% in the tables), and trained on all data from the source group and 10% data from the target group (denoted by 10% in the tables). Panels denote groups stratified by one of the four attributes, namely sex, race and clinical study. Bar plots denote the proportion of subjects in these groups in our study. All models are ensembles trained using features derived from structural measures, demographic and clinical variables, genetic factors, and cognitive scores.

**Sex**		Female	Male
0%			
	Female	-	4.6902
	Male	4.6643	-
10%			
	Female	-	4.6166
	Male	4.3896	-
80%			
	-	4.1181	4.3549

**Race**		White	Black	Asian
0%				
	White	-	4.8421	4.7436
	Black	6.1148	-	6.2676
	Asian	6.2358	6.8857	-
10%				
	White	-	4.8474	4.5357
	Black	4.5049	-	6.0057
	Asian	4.5894	5.9918	-
80%				
	-	3.9901	4.8967	5.1489

**Study**		BIOCARD	BLSA-1.5T	BLSA-3T	CARDIA	SHIP	SPRINT	UKBB	WHIMS	WRAP	lookAHEAD
0%											
	BIOCARD	-	5.1597	8.1243	5.1326	8.8638	5.0915	4.9134	3.4510	4.2352	6.4431
	BLSA-1.5T	7.0434	-	7.9681	9.1985	11.7253	5.3157	4.9993	3.1509	4.4632	9.0969
	BLSA-3T	7.5188	5.4739	-	9.7144	11.1708	6.0576	5.6380	6.5140	4.8916	9.2493
	CARDIA	8.0467	14.1256	15.3413	-	10.3559	12.8710	10.2514	15.9061	10.4533	5.2601
	SHIP	10.3542	15.3256	13.8427	6.5199	-	7.7349	14.1402	5.0092	8.9724	5.8628
	SPRINT	7.5668	6.9445	9.8383	8.5229	12.4118	-	5.8606	5.6735	4.9719	7.8257
	UKBB	6.1263	5.2981	7.5216	7.0385	11.0483	5.3614	-	3.1166	4.2923	8.1095
	WHIMS	10.3377	6.0249	10.7618	13.6363	15.3092	6.4792	6.1463	-	5.1581	7.0639
	WRAP	7.9669	5.9605	9.4942	10.1443	12.9613	5.6444	5.0151	2.8126	-	7.6354
	lookAHEAD	7.5399	12.3101	13.3466	4.2895	9.5694	11.3139	8.7010	9.1397	8.4743	-
10%											
	BIOCARD	-	4.7087	6.4790	3.0771	5.6160	4.9782	4.4169	2.7704	4.0595	4.7564
	BLSA-1.5T	5.9926	-	6.4443	3.4137	5.7878	5.0184	4.4336	2.7778	4.3133	4.7999
	BLSA-3T	5.6211	4.7634	-	3.5383	5.7111	4.6509	4.2806	2.8641	4.1537	4.5702
	CARDIA	6.7340	6.6454	7.4902	-	5.7652	5.2600	4.6932	2.8337	4.7503	4.4487
	SHIP	5.7043	5.5634	6.5381	2.9623	-	4.7351	4.4113	2.7875	4.2589	4.2730
	SPRINT	5.9473	4.8374	6.4243	3.0688	5.5828	-	4.2567	2.8009	4.1908	4.7320
	UKBB	5.3467	4.9048	5.9838	3.7585	5.6768	4.6116	-	2.7193	3.7259	4.6744
	WHIMS	6.4178	5.7135	6.7564	3.1224	5.7796	5.0562	4.4045	-	4.5165	4.6987
	WRAP	6.1384	5.5298	6.5214	3.1077	5.6488	5.1082	4.3612	2.7497	-	4.6183
	lookAHEAD	6.6080	6.3420	7.1366	3.0568	5.6129	5.3508	4.4653	2.7729	4.6745	-
80%											
	-	5.4879	4.2219	5.0418	2.7186	4.6912	4.3610	3.8118	2.6655	3.7032	3.9595

Table S.10: Mild cognitive impairment progression prediction results. Linear discriminant analysis on the output probabilities (that determines AD vs. cognitively normal CN) of the ensemble models trained for Alzheimer's disease diagnosis is used to study whether subjects with mild cognitive impairment (MCI) progress to AD (known as pMCI) or remain stable MCI (known as sMCI) using only the baseline scans. The average AUC of pMCI vs. sMCI on the target group (columns in the tables) is shown for three different attributes (sex, age group and race) when models are trained only on data from the source group (denoted by 0% in the tables), using α-weighted ERM using all data from the source and 10% data from the target group (denoted by 10% in the tables) and with access to only all data from the target group (denoted by 80% in the tables).

**Sex**		Female	Male
0%			
	Female	-	0.7312
	Male	0.8082	-
10%			
	Female	-	0.7317
	Male	0.7967	-
80%			
	-	0.7997	0.7265

**Age**		0-65	65-70	70-75	75-80	>80
0%						
	0-65	-	0.7423	0.6771	0.6570	0.5976
	65-70	0.8098	-	0.8016	0.7402	0.6260
	70-75	0.8018	0.7749	-	0.6809	0.6399
	75-80	0.8306	0.7661	0.7594	-	0.6473
	>80	0.7337	0.7355	0.7562	0.7460	-
10%						
	0-65	-	0.7551	0.7012	0.6538	0.5597
	65-70	0.7662	-	0.7622	0.7216	0.6489
	70-75	0.8230	0.7844	-	0.7152	0.6250
	75-80	0.8371	0.7639	0.7438	-	0.6558
	>80	0.8042	0.7549	0.7315	0.7099	-
80%						
	-	0.7919	0.7786	0.7594	0.7073	0.6348

**Race**		White	Black	Asian
0%				
	White	-	0.9550	1.0000
	Black	0.7118	-	0.9208
	Asian	0.5171	0.4217	-
10%				
	White	-	0.9167	1.0000
	Black	0.7257	-	0.8729
	Asian	0.6271	0.5058	-
80%				
	-	0.7759	0.9100	0.6208

Table S.11: Pearson's correlation between the brain age residual and neuropsychological tests. For two different attributes (sex and race), the models are trained only on source data (denoted by 0% in the tables), using α-weighted ERM on all source data and 10% target data (denoted by 10% in the tables) and only on all target data (denoted by 80% in the tables). Mini-mental state examination (MMSE) is a questionnaire test that measures global cognitive impairment. Digit span forward (DSF) test is a way of measuring the storage capacity of a person's working memory. Trail making test part A (TMT A) measures a person's executive functioning.

(a) Mini-mental state examination (MMSE).

**Sex**		Female	Male
0%			
	Female	-	-0.1197
	Male	-0.2865	-
10%			
	Female	-	-0.1458
	Male	-0.2674	-
80%			
	-	-0.1614	-0.0489

**Race**		White	Black	Asian
0%				
	White	-	-0.0829	-0.4625
	Black	-0.3033	-	-0.4917
	Asian	-0.2434	-0.1232	-
10%				
	White	-	-0.0777	-0.4097
	Black	-0.2974	-	-0.4609
	Asian	-0.2698	-0.1508	-
80%				
	-	-0.1447	-0.0714	-0.1495

(b) Digit span forward (DSF).

**Sex**		Female	Male
0%			
	Female	-	-0.0032
	Male	-0.0865	-
10%			
	Female	-	-0.0232
	Male	-0.0815	-
80%			
	-	0.0178	-0.0379

**Race**		White	Black	Asian
0%				
	White	-	0.0783	-0.3364
	Black	-0.1708	-	-0.3114
	Asian	-0.0113	-0.0750	-
10%				
	White	-	0.0894	-0.3134
	Black	-0.1541	-	-0.2931
	Asian	-0.0256	-0.1149	-
80%				
	-	-0.0292	-0.0780	-0.1136

(c) Trail making test part A (TMT A).

**Sex**		Female	Male
0%			
	Female	-	0.1960
	Male	0.1835	-
10%			
	Female	-	0.1940
	Male	0.1578	-
80%			
	-	0.1572	0.0476

**Race**		White	Black	Asian
0%				
	White	-	-0.0026	0.4336
	Black	0.1740	-	0.5911
	Asian	0.1279	0.0679	-
10%				
	White	-	-0.0025	0.4019
	Black	0.2101	-	0.5814
	Asian	0.1906	0.0825	-
80%				
	-	0.1157	0.2251	0.3113

Table S.12: Pearson's correlation between the brain age residual and neuropsychological tests. For two different attributes (sex and race), the models are trained only on source data (denoted by 0% in the tables), using α-weighted ERM on all source data and 10% target data (denoted by 10% in the tables) and only on all target data (denoted by 80% in the tables). Digit symbol substitution test (DSST) is another global measure of cognitive ability, requiring multiple cognitive domains to complete effectively. Trail making test part B (TMT B) measures a person's executive functioning. Digit span backward (DSB) test is a way of measuring the storage capacity of a person's working memory.

(a) Digit symbol substitution test (DSST).

**Sex**		Female	Male
0%			
	Female	-	-0.3963
	Male	-0.2232	-
10%			
	Female	-	-0.4017
	Male	-0.2001	-
80%			
	-	-0.1342	-0.1540

**Race**		White	Black	Asian
0%				
	White	-	-0.0248	-0.1814
	Black	-0.4085	-	-0.7416
	Asian	-0.4533	-0.1100	-
10%				
	White	-	-0.0227	-0.2029
	Black	-0.4266	-	-0.5764
	Asian	-0.5560	-0.1144	-
80%				
	-	-0.1191	-0.1980	-0.5151

(b) Trail making test part B (TMT B).

**Sex**		Female	Male
0%			
	Female	-	0.1979
	Male	0.2385	-
10%			
	Female	-	0.1939
	Male	0.2087	-
80%			
	-	0.1908	0.0639

**Race**		White	Black	Asian
0%				
	White	-	0.0296	0.3429
	Black	0.2377	-	0.5779
	Asian	0.1324	0.1402	-
10%				
	White	-	0.0279	0.3293
	Black	0.2808	-	0.5557
	Asian	0.2122	0.1610	-
80%				
	-	0.1191	0.1861	0.4670

(c) Digit span backward (DSB).

**Sex**		Female	Male
0%			
	Female	-	-0.0693
	Male	-0.0958	-
10%			
	Female	-	-0.0898
	Male	-0.0710	-
80%			
	-	0.0239	0.0148

**Race**		White	Black	Asian
0%				
	White	-	0.1262	-0.2682
	Black	-0.1285	-	-0.2715
	Asian	-0.0612	0.0972	-
10%				
	White	-	0.1339	-0.2306
	Black	-0.1417	-	-0.2376
	Asian	-0.0857	0.0506	-
80%				
	-	-0.0349	0.0591	0.0491
